# Supplementary material for: Menopausal symptoms, physical activity level and quality of life of women living in the Mediterranean region
Source: PLoS One. 2020 Mar 24;15(3):e0230515. doi: 10.1371/journal.pone.0230515 (PMC7093012; doi:10.1371/journal.pone.0230515)
Supplement: S1 File — (DOCX) [file pone.0230515.s001.docx]

**S1 File**

**IPAQ**

**(استبانة النشاط البدني)**

الأسئلة التالية ترتكز حول الوقت الذي قضيته في ممارسة أنشطة بدنية خلال الأيام السبعة الماضية. فكري في الأنشطة البدنية التي تمارسيها خلال عملك، وكجزء من أعمالك المنزلية، وأثناء تنقلك من مكان لآخر، وتلك التي تقوم بها في وقت فراغك.

الآن فكري في جميع الأنشطة البدنية التي تتطلب جهداً بدنياً مرتفع الشدة والتي قمت بممارستها خلال الأيام السبعة الماضية. الأنشطة البدنية **مرتفعة الشدة هي تلك الأنشطة التي تجعل تنفسك أعلى بكثير من المعتاد**، مثل رفع أشياء ثقيلة، أو ركوب الدراجة بسرعة عالية، أو الجري، أو السباحة، أو نط الحبل. فكر فقط في الأنشطة البدنية مرتفعة الشدة التي قمت بممارستها لمدة 10 دقائق على الأقل في كل مرة.

1. خلال الأيام السبعة الماضية، كم يوماً مارست فيه نشاطاً بدنياً **مرتفع الشدة**؟

____ يوم في الأسبوع

لا أقوم بأي نشاط بدني مرتفع الشدة. انتقلي مباشرة إلى السؤال رقم 3

1. في المعتاد، كم من الوقت قضيته في ممارسة نشاط بدني **مرتفع الشدة** في أحد تلك الأيام؟

____ ساعة في اليوم

____ دقيقة في اليوم

لا أدري/ أو غير متأكدة.

الآن فكري في جميع الأنشطة البدنية التي تتطلب جهداً بدنياً **معتدل الشدة** والتي قمت بممارستها خلال الأيام السبعة الماضية. الأنشطة البدنية **معتدلة الشدة هي تلك الأنشطة التي تجعل تنفسك أعلى من المعتاد إلى حداً ما**، ويمكن أن تتضمن رفع أشياء خفيفة، أو ممارسة كرة الطائرة، أو ممارسة تنس الطاولة، أو كنس المنزل. لا تحسبي المشي ضمن هذه الأنشطة. مرة أخرى، فكر فقط في الأنشطة البدنية معتدلة الشدة التي قمت بممارستها لمدة 10 دقائق على الأقل في كل مرة.

1. خلال الأيام السبعة الماضية، كم يوماً مارست فيه نشاطاً بدنياً معتدل الشدة؟

____ يوم في الأسبوع

لا أقوم بأي نشاط بدني معتدل الشدة. انتقلي مباشرة إلى السؤال رقم 5

1. في المعتاد، كم من الوقت قضيته في ممارسة نشاط بدني معتدل الشدة في أحد تلك الأيام؟

____ ساعة في اليوم

____ دقيقة في اليوم

لا أدري/ أو غير متأكدة.

الآن فكري في الوقت الذي قضيته في **المشي** خلال الأيام السبع الماضية، ويتضمن ذلك المشي إلى العمل، والمشي أثناء العمل، وفي البيت، وخلال انتقالك من مكان لآخر، أو أي نوع من أنواع المشي بغرض الترويح أو الرياضة.

1. خلال الأيام السبعة الماضية، كم يوماً مارست فيه المشي لمدة 10 دقائق على الأقل في كل مرة؟

____ يوم في الأسبوع

لا أقوم بممارسة المشي إطلاقا. انتقلي مباشرة إلى السؤال رقم 7

1. في المعتاد، كم من الوقت قضيته في ممارسة المشي في أحد تلك الأيام؟

____ ساعة في اليوم

____ دقيقة في اليوم

لا أدري/ أو غير متأكدة.

الآن فكري في الوقت الذي قضيته جالستاً خلال الأيام السبعة الماضية. أحسبي وقت الجلوس في العمل، وفي المنزل، وفي الدراسة، وفي الترفيه. من الممكن أن يتضمن ذلك وقت الجلوس على المكتب، وأثناء العمل على الكمبيوتر، وأثناء زيارتك لصديق، وأثناء القراءة، والجلوس أو الاستلقاء لمشاهدة التلفزيون.

1. خلال الأيام السبعة الماضية، كم من الوقت قضيته جالستاً في أحد هذه الأيام من غير أيام الإجازة الأسبوعية؟

____ ساعة في اليوم

____ دقيقة في اليوم

لا أدري/ أو غير متأكدة.

***(نهاية الاستبانة، شكراً لمشاركتكم)***
