# Supplementary material for: Menopausal symptoms, physical activity level and quality of life of women living in the Mediterranean region
Source: PLoS One. 2020 Mar 24;15(3):e0230515. doi: 10.1371/journal.pone.0230515 (PMC7093012; doi:10.1371/journal.pone.0230515)
Supplement: S1 Table — (DOCX) [file pone.0230515.s002.docx]

**S1 Table. Sociodemographic characteristics of the study group (N=1113).**

|  | **N** | **%** |
| --- | --- | --- |
| **Age** |  |  |
| 40 - 44 years | 248 | 22.3 |
| 45 - 49 years | 322 | 28.9 |
| 50 - 54 years | 285 | 25.6 |
| 55 - 60 years | 258 | 23.2 |
| **Place of residence** |  |  |
| Mount Lebanon | 533 | 47.9 |
| Beirut | 301 | 27 |
| Southern Lebanon | 107 | 9.6 |
| Northern Lebanon | 75 | 6.7 |
| Bekaa | 34 | 3.1 |
| Jbeil | 21 | 1.9 |
| Nabatiyeh | 19 | 1.7 |
| Baalbek | 16 | 1.4 |
| Akkar | 7 | 0.6 |
| **Educational level** |  |  |
| Elementary | 76 | 6.8 |
| Intermediate | 209 | 18.8 |
| Secondary | 318 | 28.6 |
| University degree | 510 | 45.8 |
| **Marital status** |  |  |
| Married | 909 | 81.7 |
| Single | 114 | 10.2 |
| Divorced | 45 | 4 |
| Widow | 45 | 4 |
| **Crowding index** ^ǂ^ |  |  |
| > 1 | 510 | 45.82 |
| 1 | 228 | 20.5 |
| <1 | 318 | 28.6 |
| **Profession*** |  |  |
| Employed | 500 | 45 |
| Unemployed | 582 | 52.4 |
| Retired | 29 | 2.6 |
| **Religion** |  |  |
| Muslim | 624 | 56.1 |
| Christian | 465 | 41.8 |
| Druze | 24 | 2.2 |

***^*^*** *missing values*

*^ǂ^ Crowding index: number of co-residents (excluding newborn) divided by number or rooms (excluding kitchen and bathrooms). A crowding index < 1 reflects a household with a good economical standing, 1 to an average standing and > 1 to a below average standing.*
