# Supplementary material for: Menopausal symptoms, physical activity level and quality of life of women living in the Mediterranean region
Source: PLoS One. 2020 Mar 24;15(3):e0230515. doi: 10.1371/journal.pone.0230515 (PMC7093012; doi:10.1371/journal.pone.0230515)
Supplement: S2 Table — (DOCX) [file pone.0230515.s003.docx]

**S2 Table. Clinical data according to the menopausal status of the participants (N=1113).**

| **Characteristics** | **Pre-menopause** | **Peri-menopause** | **Menopause** | **Post-menopause** | **P value** |
| --- | --- | --- | --- | --- | --- |
| **Age** | 45.3 ± 3.7 ^a^ | 48.8 ±3.0 ^b^ | 50.9 ±3.0 ^c^ | 54.3 ± 4.2 ^d^ | <0.001^*^ |
| **BMI^ǂ^** (Kg/m^2^) | 26.2 ± 5.8 ^a^ | 26.2 ± 4.1 ^a^ | 26.7 ± 4.8 ^b^ | 27.3 ± 5.1 ^c^ | 0.008^*^ |
| **Waist circumference** (cm) | 88.4 ±12.5 ^a^ | 89.9 ± 11.7 ^b^ | 90.3 ± 11.2 ^c^ | 92.4 ± 13.9 ^d^ | <0.001^*^ |
| **Hip circumference** (cm) | 101.6 ± 13.3 ^a^ | 102.9 ± 13.0 ^b^ | 104.4 ± 11.6 ^c^ | 106.1 ± 14.6 ^d^ | <0.001^*^ |
| **Waist to Hip ratio** | 0.9 ± 0.1 | 0.9 ± 0.1 | 0.9 ± 0.1 | 0.9 ± 0.1 | 0.969 |
| **Age at first period** (years) | 12.6 ± 1.5 | 12.3 ± 1.5 | 12.3 ± 1.2 | 12.6 ± 1.5 | 0.099 |
| **Age at menopause** | - | - | 49.2 ± 3.1 | 47.7 ± 4.7 | 0.012^*^ |
| **Number of children** | 2.7 ± 1.2 | 2.7 ± 1.1 | 2.7 ± 1.3 | 2.8 ± 1.4 | 0.255 |
| **Chronic diseases** | 86 (16.7%) | 18 (21.7%) | 20 (31.7%) | 156 (34.4%) | <0.001^*^ |
| *Diabetes* | 10 (1.9%) | 5 (6.0%) | 6 (9.5%) | 45 (9.9%) | <0.001^*^ |
| *Hypertension* | 43 (8.4%) | 8 (9.6%) | 9 (14.3%) | 95 (21.0%) | <0.001^*^ |
| *Cardiovascular disease* | 6 (1.2%) | 4 (4.8%) | 1 (1.6%) | 26 (5.7%) | <0.001^*^ |
| **Smoking** | 236 (45.9%) | 43 (51.8%) | 24 (38.1%) | 263 (58.1%) | <0.001^*^ |
| **Alcohol consumption** | 147 (28.6%) | 23 (27.7%) | 23 (36.5%) | 136 (30.0%) | 0.472 |
| **Coffee consumption** | 432 (84.0%) | 76 (91.6%) | 59 (93.7%) | 415 (91.6%) | <0.001^*^ |
| **Regularity of menstrual cycles** | 447 (87.0%) | 70 (84.3%) | 52 (82.5%) | 372 (82.1%) | 0.209 |
| **Increase in appetite** | 216 (42.0%) | 51 (61.4%) | 35 (55.6%) | 198 (43.7%) | 0.003^*^ |
| **Self-evaluation of health** |  |  |  |  |  |
| *Excellent/Good* | 353 (68.7%) | 37 (44.6%) | 38 (60.3%) | 256 (56.5%) | <0.001^*^ |
| *Normal* | 147 (28.6%) | 34 (41.0%) | 23 (36.5%) | 163 (36.0%) |  |
| *Bad/Very bad* | 14 (2.7%) | 12 (14.5%) | 2 (3.2%) | 34 (7.5%) |  |

^ǂ^*BMI: body mass index ; ^*^Statistical analyses were done with ANOVA and Chi-square tests with a p value <0.05 considered as significant.*

*a, b, c, d: different letters indicate the presence of significant letters with Tukey post hoc tests; mean ±SD with letter “a” have the significant lowest values, and letters “d” indicate the highest values*
