# Supplementary material for: Menopausal symptoms, physical activity level and quality of life of women living in the Mediterranean region
Source: PLoS One. 2020 Mar 24;15(3):e0230515. doi: 10.1371/journal.pone.0230515 (PMC7093012; doi:10.1371/journal.pone.0230515)
Supplement: S3 Table — (DOCX) [file pone.0230515.s004.docx]

**S3 Table. The distribution of the menopausal symptoms according to the menopausal status (N=1113).**

|  | | **Menopausal status** | | | |  | |
| --- | --- | --- | --- | --- | --- | --- | --- |
|  |  | Pre- menopause  N = 514 | Peri- menopause  N = 83 | Menopause  N = 63 | Post-menopause  N = 453 | **Total** | **P value** |
| **Vasomotor subdomain** | Hot ﬂashes | 168 (32.7%) | **54 (65.1%)** | 38 (60.3%) | 284 (62.7%) | 544 (48.9%) | <0.001^*^ |
|  | Night sweats | 124 (24.1%) | **42 (50.6%)** | 29 (46.0%) | 208 (45.9%) | 403 (36.2%) | <0.001^*^ |
|  | Sweating | 51 (9.9%) | 18 (21.7%) | 13 (20.6%) | **118 (26.0%)** | 200 (18.0%) | <0.001^*^ |
| **Psychosocial**  **subdomain** | Being dissatisfied with my personal life | 197 (38.3%) | 44 (53.0%) | 22 (34.9%) | 182 (40.2%) | 445 (40.0%) | 0.068 |
|  | Feeling anxious or nervous | 351 (68.3%) | 65 (78.3%) | 45 (71.4%) | 306 (67.5%) | 767 (68.9%) | 0.251 |
|  | Experiencing poor memory | 254 (49.4%) | 51 (61.4%) | 31 (49.2%) | 253 (55.8%) | 589 (52.9%) | 0.076 |
|  | Accomplishing less than i used to | 188 (36.6%) | **49 (59.0%)** | 27 (42.9%) | 219 (48.3%) | 483 (43.4%) | 0.001^*^ |
|  | Feeling down or depressed | 212 (41.2%) | 43 (51.8%) | 22 (34.9%) | 187 (41.3%) | 464 (41.7%) | 0.191 |
|  | Being impatient with other people | 252 (49.0%) | **53 (63.9%)** | 27 (42.9%) | 218 (48.1%) | 550 (49.4%) | 0.039^*^ |
|  | Feelings of wanting to be alone | 221 (43.0%) | **45 (54.2%)** | 22 (34.9%) | 175 (38.6%) | 463 (41.6%) | 0.034^*^ |
| **Physical subdomain** | Flatulence or gas pain | 276 (53.7%) | 46 (55.4%) | 37 (58.7%) | 252 (55.6%) | 611 (54.9%) | 0.854 |
|  | Aching in muscles and joints | 344 (66.9%) | **63 (75.9%)** | **46 (73.0%)** | **352 (77.7%)** | 805 (72.3%) | 0.002^*^ |
|  | Decrease in physical strength | 248 (48.2%) | **55 (66.3%)** | 33 (52.4%) | 263 (58.1%) | 599 (53.8%) | 0.002^*^ |
|  | Aches in back of neck or head | 349 (67.9%) | 68 (81.9%) | 42 (66.7%) | 317 (70.0%) | 776 (69.7%) | 0.073 |
|  | Difficulty in sleeping | 223 (43.4%) | **51 (61.4%)** | 28 (44.4%) | 240 (53.0%) | 542 (48.7%) | 0.002^*^ |
|  | Feeling tired or worn out | 370 (72.0%) | 63 (75.9%) | 39 (61.9%) | 341 (75.3%) | 813 (73.0%) | 0.124 |
|  | Decrease in stamina | 232 (45.1%) | **52 (62.7%)** | 27 (42.9%) | 218 (48.1%) | 529 (47.5%) | 0.024^*^ |
|  | Feeling a lack of energy | 283 (55.1%) | **56 (67.5%)** | 34 (54.0%) | 280 (61.8%) | 653 (58.7%) | 0.049^*^ |
|  | Dry skin | 256 (49.8%) | 44 (53.0%) | 30 (47.6%) | 211 (46.6%) | 541 (48.6%) | 0.634 |
|  | Weight gain | 268 (52.1%) | **59 (71.1%)** | 38 (60.3%) | 260 (57.4%) | 625 (56.2%) | 0.009^*^ |
|  | Increased facial hair | 90 (17.5%) | 16 (19.3%) | 6 (9.5%) | 88 (19.4%) | 200 (18.0%) | 0.276 |
|  | Changes in appearance, texture or tone of skin | 151 (29.4%) | 30 (36.1%) | 20 (31.7%) | 165 (36.4%) | 366 (32.9%) | 0.118 |
|  | Feeling bloated | 260 (50.6%) | 53 (63.9%) | 36 (57.1%) | 232 (51.2%) | 581 (52.2%) | 0.119 |
|  | Low backache | 310 (60.3%) | **65 (78.3%)** | 43 (68.3%) | 306 (67.5%) | 724 (65%) | 0.005^*^ |
|  | Frequent urination | 141 (27.4%) | **37 (44.6%)** | 23 (36.5%) | 170 (37.5%) | 371 (33.3%) | 0.001^*^ |
|  | Involuntary urination when laughing or coughing | 114 (22.2%) | 28 (33.7%) | 19 (30.2%) | 113 (24.9%) | 274 (24.6%) | 0.092 |
| **Sexual**  **Subdomain** | Modification in sexual desire | 177 (35.4%) | 40 (48.2%) | **33 (53.2%)** | 221 (50.1%) | 471 (43.4%) | <0.001^*^ |
|  | Vaginal dryness during intercourse | 102 (20.4%) | 29 (34.9%) | **26 (41.9%)** | 153 (34.7%) | 310 (28.5%) | <0.001^*^ |
|  | Avoiding intimacy | 149 (29.8%) | 34 (41.0%) | 23 (37.1%) | **197 (44.7%)** | 403 (37.1%) | <0.001^*^ |

*^*^Statistical analysis were done with Chi-square tests, with a p value <0.05 considered as significant*
