# Supplementary material for: Menopausal symptoms, physical activity level and quality of life of women living in the Mediterranean region
Source: PLoS One. 2020 Mar 24;15(3):e0230515. doi: 10.1371/journal.pone.0230515 (PMC7093012; doi:10.1371/journal.pone.0230515)
Supplement: Attachment — Submitted filename: Review_PONE-D-19-25613.docx [file pone.0230515.s005.docx]

**PONE-D-19-25613**

**Comments to the authors**

I appreciate the effort of the authors to conduct the study for reporting the relationship between physical activity level, menopausal symptoms and the quality of life of Lebanese women. After completling my review, several concerns should be revised to fulfill the quality of the study.

**General Comments**

Please standardize the objective of the study on the different topics of this article (i.e. abstract, introduction and, discussion).

Please review your reference as more than half of the references listed are very outdated.  If able, prefer to keep references not older than 10 years.

In addition, some English mistakes need to be corrected through all the manuscript.

**Specific Comments**

**Abstract**

In methods, please provide information regarding the classification of menopausal status.

**Introduction**

Please add bibliographic references in the first sentences of the first paragraph.

Please add the meaning of “MENQQOL”.

**Methods**

Please provide information regarding the randomization process of the different regions recruited.

Was any participant using hormone replacement therapy?

The authors do not mention the application of a cognitive test. Participants with cognitive impairment may have difficulty with the questionnaires.

**Results**

Please review this session. Some table results are not consistent with the text.

Please review the table captions. It is not possible to identify statistical differences among/between groups.

Please add to table 4 the results of the physical activity level data analysis.

**Discussion**

1° Paragraph: Please discuss more about the impact of anthropometric measurements (e.g. BMI, WC and WHR) on participants of this current study. Or join this part with the beginning of the 4° paragraph.

Please discuss more about the relationship between the results of the activity physical level and the different subdomains of women's quality of life.

Please discuss whether the results reach the level of clinical significance.
