# Supplementary material for: Menopausal symptoms, physical activity level and quality of life of women living in the Mediterranean region
Source: PLoS One. 2020 Mar 24;15(3):e0230515. doi: 10.1371/journal.pone.0230515 (PMC7093012; doi:10.1371/journal.pone.0230515)
Supplement: Attachment — Submitted filename: Rebuttal letter MENQOL.docx [file pone.0230515.s006.docx]

Dear editor and reviewers,

On behalf of all the authors, we highly appreciate your valuable comments and recommendations, that were fully taken into consideration in the final revised submitted manuscript. Hope it will meet your requirements.

Journal Requirements:

1. When submitting your revision, we need you to address these additional requirements. Please ensure that your manuscript meets PLOS ONE's style requirements, including those for file naming. The PLOS ONE style templates can be found at <http://www.journals.plos.org/plosone/s/file?id=wjVg/PLOSOne_formatting_sample_main_body.pdf> and<http://www.journals.plos.org/plosone/s/file?id=ba62/PLOSOne_formatting_sample_title_authors_affiliations.pdf>

Done.

2.  Please include captions for your Supporting Information files at the end of your manuscript, and update any in-text citations to match accordingly. Please see our Supporting Information guidelines for more information: <http://journals.plos.org/plosone/s/supporting-information>.

Done. Two supplementary tables were added and mentioned in the text.

3. Please clarify in your Methods section whether the questionnaire is published under a CC-BY license, or whether you obtained permission from the publisher to reproduce the questionnaire in this manuscript. Please explain any copyright or restrictions on this questionnaire

After conducting the literature review and before proceeding, permission was solicited from the research team* (Dr Luma el Zamil/lumazamil@yahoo.com) that applied this self-administered tool in its Arabic version, in a study among Saudi women. Dr Zamil requested only to cite her article in the reference section (Ref 12 in our article).

*Mohamed HA, Lamadah SM, Zamil LG. Quality of life among menopausal women. Int J Reprod Contracept Obstet Gynecol 2014;3:552-61.

4. Please change your reference to "p=0.000" to "p<0.001" or as similarly appropriate, as p values cannot equal zero.

Done. All p values were corrected from p=0.000 to p<0.001.

5. Please amend your manuscript to include your abstract after the title page.

Done.

6. Please ensure that you include a title page within your main document. You should list all authors and all affiliations as per our author instructions and clearly indicate the corresponding author.

Done.

Review Comments to the Author

Please use the space provided to explain your answers to the questions above. You may also include additional comments for the author, including concerns about dual publication, research ethics, or publication ethics. (Please upload your review as an attachment if it exceeds 20,000 characters)

Reviewer #1: I read with great interest the study "Menopausal symptoms, physical activity level and quality of life of women living in the
Mediterranean region" by Papazian et al.

The paper is well written, and the study deals with an important topic (i.e., menopause and quality of life), which has been extensively studied in western populations, but not too much in the Middle-East. Since there might be cultural- and social-related differences the way women cope with menopause, I think the current study is original in the sense that it originally provides a large amount of updated data related to quality of life, physical activity, anthropometrics in a rather understudied population.

The authors should be praised for their hard work and I have only minor questions/suggestions for improvements.

1) In introduction I would change "often failed to fully describe" to " a less strong sentence (maybe "present difficulties in accurately describing").
Done.

2) It is not clear if "hormone replacement therapy" was/wasn't a exclusion criteria.
Hormone replacement therapy was an exclusion criteria.

3) I feel sorry for the 415 questionnaires that had to be removed from the final pool due to incomplete data. However, I wonder if the researchers couldn't at least compute the QOL domains that were filled by the participants and not exclude the whole data.
Unfortunately , the removal of some of the questionnaires from the final pool was not well elucidated in our initial version of the manuscript. In fact, the research director distributed around 1528 questionnaire among the research team to approach eligible candidates. Some women were either reluctant to join, non-Lebanese, born Lebanese but living abroad, or having difficulties in reading Arabic. Hence, statistical analysis were performed on the total number of complete collected material (N=1113). This issue is clarified in the final revised manuscript (L. 129).

4) I also wonder if excluding the women that did not answer sexual-related questions couldn't be faced as a bias of the study. Couldn't part of the results of these women (the one that they answered) be included in the final analysis?

From the final pool of the sample (N=1113), only 27 women were reluctant to answer questions related on sexual issues, but filled all the other parts of the questionnaires. Their data was included. They represent 2.4% of the total participants.

5) Overall, I don't think it is necessary to explain in details all the questionnaires (e.g., lines 70-77 for MENQOL; lines 80-81 for IPAQ, and so on). Authors should keep it simple and provide references for further details.

Done. We summarized this section.

6) Tables 1 and 2 could be merged.

Done.

7) On all Tables authors should make it very clear the meaning/role of the overwritten letters.

Done.

8) Discussion on lines 232-243 is not necessary as it does not related to the objective of the study.
We aimed to present this information and compare it with other published data, since our study is the first conducted among a large number of women, and no national statistics were available.

9) I think the physical activity is a nice outcome of the study, and this was independently associated with most of the subdomains of quality of life in the participants, which reinforce the importance of this variable to the study conclusions. However, authors should acknowledge the limitations of using a questionnaire to assess physical activity as it might present inaccuracies in comparison with objective measurements (e.g., accelerometers).
Done. This issue was mentioned in the limitation section.

10) Another limitation is the absence of blood tests to check levels of FSH and estrogen hormones (even if it was done in a sub sample). I would include this in the limitation section but I don't think this limitation reduce the quality of the study.
Blood tests were not included in the methodology of this study. A sentence was added in the limitation section concerning this issue.

11) It is not clear if there was a sampling strategy (and sample calculation) to generate a sample representative of all Lebanon areas. If not, I would include this as a limitation of the study.

The minimum sample size to be included in this study was calculated initially by following the formula of Tabachnick and Fidell^1^ (2001) that take into consideration the number of explanatory variables to be include in the model: N = 50 + 8m (m is the number of explanatory variables); Given that m=14, a minimum of 162 women should be recruited in this study. Our sampling strategy was not representative of all Lebanese women since we followed a convenient sampling strategy, however it englobed a large number of participants from different educational, marital and religious backgrounds and of various stages of the menopausal transition.This explanation was added in the final manuscript.

^1^Tabachnik BG, Fidell LS. Using multivariate statistics. 4th ed. Needham Heights, New York: Harper Collins (2001)

Reviewer #2: I appreciate the effort of the authors to conduct the study for reporting the relationship between physical activity level, menopausal symptoms and the quality of life of Lebanese women. After completling my review, several concerns should be revised to fulfill the quality of the study.

General Comments
Please standardize the objective of the study on the different topics of this article (i.e. abstract, introduction and, discussion).

Done

Please review your reference as more than half of the references listed are very outdated. If able, prefer to keep references not older than 10 years.

Done. We removed 6 old references and added 14 new ones.

In addition, some English mistakes need to be corrected through all the manuscript.
The manuscript was corrected by a colleague, whose native language was English.

Specific Comments
Abstract
In methods, please provide information regarding the classification of menopausal status.
Done. We clarified this classification in the text by following WHO’s division.
Introduction
Please add bibliographic references in the first sentences of the first paragraph.

Done. New References were added to the final text in the introduction, such as:

1. Maric-Bilkan C, Gilbert EL, Ryan MJ. Impact of ovarian function on cardiovascular health in women: focus on hypertension. Int J Womens Health. 2014;6:131–9.

2. Cauley JA. Estrogen and bone health in men and women. Steroids. 2015 Jul;99(Pt A):11–5.

3. Bruce D, Rymer J. Symptoms of the menopause. Best Pract Res Clin Obstet Gynaecol. 2009 Feb;23(1):25–32.

Please add the meaning of “MENQQOL”.

Done. We specified the meaning of Menqol in the final manuscript.

Methods
Please provide information regarding the randomization process of the different regions recruited.

Our sampling strategy was not representative of all Lebanese women since we followed a convenient sampling strategy, however it englobed a large number of participants from different educational, marital and religious backgrounds and of various stages of the menopausal transition. Data emerged from this study can help researches establish future perspectives on national level by applying proper randomization and stratification and adding more parameters. This sentence was added in the limitation section.

Was any participant using hormone replacement therapy?

Women taking hormone replacement therapy were excluded during recruitment.

The authors do not mention the application of a cognitive test. Participants with cognitive impairment may have difficulty with the questionnaires.
Participants suffering from mental disorders such as dementia or cognitive impairment were not invited to participate in the study. Those medical conditions were considered an exclusion criteria, during recruitment.

Results
Please review this session. Some table results are not consistent with the text.

Done. All tables in the result section were corrected and arranged.

Please review the table captions. It is not possible to identify statistical differences among/between groups.
Done. All table captions were corrected an arranged.

Please add to table 4 the results of the physical activity level data analysis.
Done. The results were added.

Discussion
1° Paragraph: Please discuss more about the impact of anthropometric measurements (e.g. BMI, WC and WHR) on participants of this current study. Or join this part with the beginning of the 4° paragraph.

Done. We joined this part with the beginning of the 4^th^ paragraph.

Please discuss more about the relationship between the results of the activity physical level and the different subdomains of women's quality of life.

Done. We added a new paragraph discussing this relationship (L.815-844).

Please discuss whether the results reach the level of clinical significance.

As presented in Table 5, highly significant statistical results were observed between the physical activity level and the four domains of the MENQOL questionnaire (vasomotor, psychosocial, physical and sexual). Values obtained between the domains and the categorization of physical activity as low, moderate and high presented a good and acceptable level of clinical significance, explained by the fact that women who were physically more active, were experiencing less discomforts in all the tested domains.
