# Supplementary material for: Menopausal symptoms, physical activity level and quality of life of women living in the Mediterranean region
Source: PLoS One. 2020 Mar 24;15(3):e0230515. doi: 10.1371/journal.pone.0230515 (PMC7093012; doi:10.1371/journal.pone.0230515)
Supplement: Attachment — Submitted filename: Review_PONE-D-19-25613R1.docx [file pone.0230515.s007.docx]

**PONE-D-19-25613R1**

**Comments to the Author**

In their majority, comments and suggestions made in the first round of the peer review process have been attended. Minor review is still needed to improve the manuscript.

**Abstract**

Please remove the word “randomly”, since patient recruitment was not randomized.

Please change “postmenopausal in the sexual domain (p<0.001).” to “post-menopausal and menopausal women in the sexual domain (p<0.001)”.

**Results**

It is still difficult for the reader to understand the statistical differences among / between groups in the tables. I suggest a review them.

I suggest adding a table with the clinical data (e.g. anthropometric measurements, lifestyle, health) according to the menopause status.
